# Influencing resilience: the role of policy entrepreneurs in mainstreaming climate adaptation

**DOI:** 10.1111/disa.12338

**Published:** 2019-04-04

**Authors:** Thomas Tanner, Rizwan Uz Zaman, Sunil Acharya, Elizabeth Gogoi, Aditya Bahadur

**Affiliations:** ^1^ Reader in Environment and Development at the Centre for Development, Environment and Policy, SOAS University of London United Kingdom; ^2^ Assam State Team Leader, Action on Climate Today India; ^3^ Project Manager at Practical Action Nepal Nepal; ^4^ Consultant at Oxford Policy Management India; ^5^ Research Associate at the Overseas Development Institute United Kingdom

**Keywords:** adaptation, mainstreaming, policy influence, policy processes, policy entrepreneurs, resilience

## Abstract

One way to make development pathways more resilient in the face of a changing climate has been through mainstreaming adaptation into government policies, planning and sectoral decision‐making. To date, many of the transferable lessons have taken the form of technical approaches such as risk assessments and toolkits. This article instead draws on evidence from South Asia to emphasise some of the more tacit and informal approaches used to influence adaptation policy. Despite their apparent significance in policy processes, such tactics are often neither planned for nor well reported in resilience‐building projects and programme documents. Using evidence to populate a typology of influencing strategies, this article looks particularly at the role of policy entrepreneurs who navigate the political complexity of both formal and informal governance systems to promote successful adaptation mainstreaming. It concludes with recommendations for adaptation and resilience programming that can more effectively harness the breadth of influencing strategies.

## Introduction

There is growing awareness of the need for government policy and planning to enhance resilience by adapting to the changing climate, fuelled by international events such as the 2015 Paris Climate Summit and periodic media interest following climate‐related disaster events. Acknowledging the inherent inter‐relationships between adaptation and development processes, many efforts attempt to integrate or ‘mainstream’ climate change into development planning and sectoral decision‐making (Mitchell et al., 2009; Bickersteth et al., 2017). Adaptation measures thereby become ‘part of a broader suite of measures within existing development processes and decision cycles’ (OECD, 2009, p. 60).

Mainstreaming may be horizontal, where climate objectives are compatible with policies and mechanisms across sectors (such as agriculture, water or health), or vertical across government hierarchies (international, national, sub‐national). Such integration is supported because it can coordinate across actions across different sectors and scales and ensure the long‐term sustainability of investments, and is seen as more efficient and effective than designing, implementing and managing climate policy separately from ongoing development efforts (Persson and Klein, 2008).

Many of the transferable lessons on mainstreaming to date relate to technical approaches to supporting mainstreaming, such as providing training and preparing risk assessments and toolkits. Yet the literature on political economy and policy processes also underlines the importance of understanding adaptation processes in terms of the more informal, tacit processes that are involved (Court and Maxwell, 2005; Birkmann et al., 2010; Naess et al., 2015). This article combines perspective and theories on policy‐influencing from the literature with empirical evidence from the Action on Climate Today (ACT) programme in South Asia, a five‐year initiative that works closely with governments to develop strategies to build resilience to the impact of climate change.

As such, the article looks in particular at the processes of policy‐influencing employed by externally financed technical support programmes such as ACT, testing the results by populating a transferable typology of influencing strategies. While the line between formal and informal strategies is blurred, the analysis draws particular attention to more informal strategies and mechanisms that can inform governments’ response to assessing and managing climate risk. Despite their apparent significance in policy processes, these informal tactics are often neither planned for nor well reported in resilience‐building projects and programme documents. Finally, the article provides lessons and guidance for structuring technical assistance support to optimise the potential for policy influence and for securing ownership of adaptation by governments.

This article will be of interest to all those seeking to influence policy on climate change adaptation, and in particular to those designing and providing technical assistance to support national and sub‐national governments to mainstream adaptation into their development policies.

## Influencing resilience policy: mainstreaming climate adaptation

### Mainstreaming adaptation: Politics and challenges

This article sets the mainstreaming process within a growing critique of the technical framing of adaptation. By externalising climate hazards from the natural and human systems that generate them, objective science is privileged in informing adaptation without challenging the political and economic drivers of climate change (Brown, 2015). Countering this technical framing, there is growing acknowledgement of the ways that climate change is constructed socio‐politically, such that the different conceptualisations of the problem are influenced by political processes, which in turn determine which (and whose) knowledge and interests are represented and which are excluded (Hulme, 2010; Taylor, 2014; Naess et al., 2015; Nightingale, 2017).

The choice of adaptation actions to take is therefore governed by political negotiation and mediated by existing power relations (Naess et al., 2015). These actions create trade‐offs that may favour some (usually weaker) groups at the expense of other (usually more powerful) groups, such as when flood protection moves flood water to poorer communities elsewhere (Bahadur and Tanner, 2015; Atteridge and Remling, 2018). Technically‐led approaches may also mask governance through informal and tacit policy processes, and limit the attention paid to fluid forms of power (such as negotiation) and knowledge (such as learning) (Vink et al., 2013).

The cross‐sector complexity of adaptation requires the involvement of multiple and overlapping institutions and actors with a wide range of multi‐scalar entry‐points for mainstreaming. Yet rules of business and institutional incentive structures may be poorly aligned to deal with this challenge, and human capacities may not be equipped for cross‐sector and interdisciplinary working. While emerging polycentric options for climate governance are being explored to help overcome these challenges, they remain largely untested (Jordan et al., 2015). Additionally, despite the arguments in favour of mainstreaming, in many cases adaptation has been implemented as a more discrete set of projects and programmes. This reflects the project‐oriented nature of both international funding models and many government departments leading on climate change in the Global South, but also by the widespread desire to learn how climate adaptation and building resilience is different from business‐as‐usual development (Yamin, 2005).

### Formal and informal policy influence: the role of policy entrepreneurs

Policy influence in the context of this article is concerned with enhancing the processes of adaptation mainstreaming: embedding climate adaptation concerns within government policies, budgets and decision‐making structures. It is assumed to be a positive influence, seeking action on adaptation rather than attempting to block action on climate change. Policy influence is particularly important in the context of externally supported programmes that seek to secure government ownership and support for adaptation mainstreaming. In such programmes and efforts, the ‘process’ involved in the intervention (how it is done) is as important as what activities are carried out.

Climate change impacts and the need for adaptation have traditionally been communicated through scientific assessments, which can confuse non‐technical experts and lead to predominantly technical solutions (Klein et al., 2007; Eriksen et al., 2015). The strategies for policy influence in externally financed adaptation mainstreaming programmes reflect this tendency, and are commonly formalised into technical steps (OECD, 2009; UNDP and UNEP, 2011). These follow activities to deliver a set of prescribed outputs or outcomes, such as developing new policies, implementing new activities or demonstrating changes in decision‐making processes. The common model therefore typically involves providing technical assistance to a government department that can generate analysis of climate impacts and vulnerability in key sectors (such as agriculture); development of policies and options for adaptation (such as new seed varieties); and translating these into a workplan (such as training of extension services). Policy influence is sought by providing support to decision‐making processes; producing new information and knowledge; and strengthening institutional capacity through training and advice and making links with other policy processes, such as the UN climate change regime.

This scientific approach supports a linear view of the policy process, in which evidence is brought to bear on policy. However, in combination with these ‘formal’ technical approaches, mainstreaming efforts often employ more informal and tacit strategies. While toolkits and analytical methodologies exist for formal and technical strategies to support mainstreaming, these informal strategies are far less codified, shared or explicitly written into programming, despite their potential importance (Court and Maxwell, 2005; Fraser and Kirbyshire, 2017).

To some extent, the distinction between formal and informal influencing strategies parallels that between formal institutions (the written constitution, laws, policies, file notes, rights and regulations enforced by official authorities) and informal institutions (the (usually unwritten) social norms, customs or traditions that shape thought and behaviour) (Unsworth, 2010). Practitioners and scholars alike are giving greater emphasis to the political dimensions of climate change, where informal interactions may be as important as formal governance in determining whether mainstreaming is successful (Tanner and Allouche, 2011; Taylor, 2014; Paterson and P‐Laberge, 2018). Development agencies are now acknowledging the contextual politics of the government planning and policy‐making process, and the power relations between different groups within them (World Bank, 2017).

One way of understanding the processes of policy influence examines the role of individuals who are able to navigate the political complexity of both formal and informal systems of governance. Such individuals have been referred to as ‘policy entrepreneurs', defined as ‘political actors who seek policy changes that shift the status quo in given areas of public policy’ (Mintrom, 2015, p. 103). They advocate new ideas and develop proposals; define and reframe problems; specify policy alternatives; broker ideas among the many policy actors; mobilise public opinion; and help set the decision‐making agenda (Roberts and King, 1991; Mintrom and Norman, 2009). According to John Kingdon (1984, p. 122), similar to a business entrepreneur, their defining characteristic ‘is their willingness to invest their resources – time, energy, reputation, and sometimes money – in the hope of a future return'.

Policy entrepreneurs understand political contexts and look for political opportunities to present new ideas in new ways (Mintrom and Vergari, 1996). To do so, they combine the formal strategies familiar to technical approaches to mainstreaming and a set of more tacit, informal strategies. The approach to policy entrepreneurship taken will depend on the personalities of both the influencer and those being influenced. Some are more comfortable using scientific evidence as the main pillar of influencing strategies; others may draw on personal rapport; others still may favour using networks and political incentives for policy change. Crucially, a policy entrepreneur is able to make arguments and strategies that break down traditional alignments of interests. This deep engagement with the policy space enables strategic thinking about who might support and oppose a particular change (Taylor, 2005).

This article employs the concept of policy entrepreneurship to explore strategies for adaptation policy influence while being mindful of its limitations. Evidence elsewhere suggests that, while policy entrepreneurship may be useful to establish collaborations and plans, its role is temporary, bringing into question the sustainability of such an approach (Faling et al., 2018). Conceptualising policy entrepreneurship as a means to an end to deliver policy influence may also divert attention from understanding the policy entrepreneur as an advocate with subjective goals (Kingdon, 1984).

Rather than being purely responsive to evidence and policy contexts, different entrepreneurs may be promoting particular variants of adaptation, or concerns particular to their experience or training. This cautions against projecting neutrality in the ‘future return’ for policy entrepreneurs referred to by Kingdon above. As such, evidence within this process is not neutral and is a political resource that may or equally may not help further entrepreneurial plans (Saxena, 2005; Lucas, 2018). While promoting a common policy cause such as adaptation mainstreaming might be viewed as collective entrepreneurship of multiple actors, it might also be characterised by conflicting activities (Faling et al., 2018).

Similarly, as well as promoting certain policy, institutional or decision‐making characteristics, influencing is focused on preventing negative processes and outcomes. Despite this, there is little discussion in the literature on policy processes around ‘negative lesson‐drawing', or learning what not to do (Dunlop, 2017; Stone, 2017). Policy entrepreneurs are well placed to understand why policies may fail and to facilitate policy learning—a process that both practitioners and researchers have largely overlooked.

Taking on board the limitations of the approach, the following section draws on insights from the literature along with empirical evidence on policy influence from the ACT programme to propose a typology of influencing strategies that can be employed by those seeking to promote adaptation mainstreaming in South Asia and elsewhere.

## A typology of policy‐influencing strategies

The complexity of the policy system and related influencing approaches defies easy classification or replicable toolkits. Different influencing strategies may be deployed simultaneously, with informal actions as catalytic adjuncts to the more formalised processes supporting adaptation mainstreaming. Combining academic insights and the ACT experience has enabled us to share some lessons, generated by means of a typology of influencing strategies. The typology builds on Kingdon (1984) and Roberts and King's (1991) concept of a public or policy entrepreneur, an initial typology developed by Simon Maxwell, as outlined in Start and Hovland's (2004) policy‐influencing toolkit and drawn upon by the Research and Policy in Development (RAPID) Outcome Mapping Approach (ROMA) toolkit (Young et al., 2015). Each area of the typology in turn draws on a range of other conceptual foundations, as explained briefly below. This classification helps us to understand and share knowledge on the strategies employed to influence policy around adaptation, beyond the ‘formal’ approaches.

One of the principal ways that practitioners, bureaucrats and policy‐makers articulate and make sense of complex realities is through simplified stories or scenarios known in the literature as ‘policy narratives’ (Roe, 1991). Literature on environmental policy narratives demonstrates that these can sometimes be profoundly misleading but they are nevertheless very powerful (Leach and Mearns, 1996). Powerful stories can help set norms (such as the responsibility of government to factor in climate change impacts and adaptation), as well as to communicate and convince policy‐makers of problems and the range of potential solutions. Importantly, ‘counternarratives’ can emerge that support certain interests or priorities, often linked to the *status quo* in the face of disruptive change. For adaptation, the narrative around ‘climate‐resilient development’ has commonly emphasised protecting development progress and the co‐benefits of risk management even in the absence of climate change or disaster events (Global Commission on the Economy and Climate, 2014; Surminski and Tanner, 2016).

Second, building rapport and gaining and maintaining trust is an important component of partnerships with government authorities, both political and bureaucratic, as well as other actors, networks and coalitions. There is much evidence on the importance of governments and other actors having trust in climate information and data (Ziervogel et al., 2005; Tall et al., 2014). Work on policy entrepreneurship also suggests that the ability to influence policy has built on mutual trust, respect or friendship. Equally, ‘when people feel angry, offended, annoyed, or betrayed, negotiations can be very difficult’ (Brouwer and Biermann, 2011, p. 6). Activities undertaken in order to build rapport and trust may be informal and opportunistic. Trust is also vital for maintaining networks, coalitions and cooperation across the different scales, actors and sectors of the mainstreaming process.

Third, policy‐making usually takes place within communities and networks of people who know and interact with each other. The increasingly information‐ and data‐driven economy has increased the power of networks structuring society (Castells, 1996). The role of knowledge networks in shaping policy is therefore increasingly important, not least given the ability of climate change impacts and adaptation to span geographical borders (Maxwell and Stone, 2004). Networks can act as ‘bridging organisations’ between government and non‐government spheres and across sectors (Folke et al., 2005). Policy entrepreneurs working on mainstreaming interventions are also well placed to broker and facilitate coalitions and networks across formal and informal actors and spaces. Governments use shadow networks—informal spaces of information and knowledge exchange—to introduce and sustain new ideas (Leck and Roberts, 2015). Bearing the costs of convening and brokering new agreements, including supporting spaces for interaction and information‐sharing, can provide an effective way to foster institutional reform (Fraser and Kirbyshire, 2017).

Fourth, policy change often occurs through individual leaders who develop and carry through the vision of change. These individuals (and groups of individuals) can identify politically intelligent routes through the bureaucracy and government machinery, as well as bring others on board with a common vision. In turn, they provide nodal points for those seeking to influence change. Champions can be identified and nurtured through adaptation mainstreaming interventions, and they themselves then become the policy entrepreneurs. They bring ideas and issues to the policy environment, initiating institutional and policy change by leveraging their positions and resources to achieve desired outcomes (Carmin and Anguelovski, 2012).

Leadership is increasingly acknowledged as important to address the challenges of climate adaptation by being able to (Meijerink and Stiller, 2013) (1) influence the policy process so as to get adaptation policies accepted and implemented; (2) enhance connectivity across different policy‐making levels, sectors and actors; (3) enhance the capacity of society to learn in response to feedback from the natural system and to anticipate long‐term impacts of climate change; (4) increase the adaptive capacity (adaptability) of governance networks concerned with climate adaptation.

However, there are risks in relying on individual leaders and champions. Such individuals may suddenly move post, leaving a vacuum, or may highlight and push for one kind of problem definition over another (Roberts and King, 1991). Diversity may be one way of mitigating such risks. Successful adaptation approaches elsewhere have noted the importance of involving champions and policy entrepreneurs who span both formal (e.g. government officials) and informal (such as volunteers and community leaders) arenas (Meijerink and Stiller, 2013; Bahadur and Tanner, 2014).

Finally, changing policy does not always equate to change on the ground. This is especially common in externally supported interventions such as the adaptation mainstreaming context analysed in this article. As such, there can be a significant implementation gap between what politicians and policy‐makers think they are doing and what actually happens. Influencing tactics can therefore engage not just with senior level policy‐makers but also with what Lipsky (2010) termed the ‘street‐level bureaucrats'. Working to influence these change agents and implementers acknowledges the complexity of decision‐making and the influence of a variety of top‐down and bottom‐up causes and processes in adaptation decision making (Biesbroek et al., 2015). The street‐level bureaucrats have the functional power to influence decisions through, for example, the interpretation of guidelines, the undertaking of performance evaluation, the solving of problems and the actual implementation of governance mechanisms (Morrison et al., 2017). These powers help explain why changes to formal rules and regulations do not always lead to the intended outcomes (Antonson et al., 2016).

**Figure 1:**
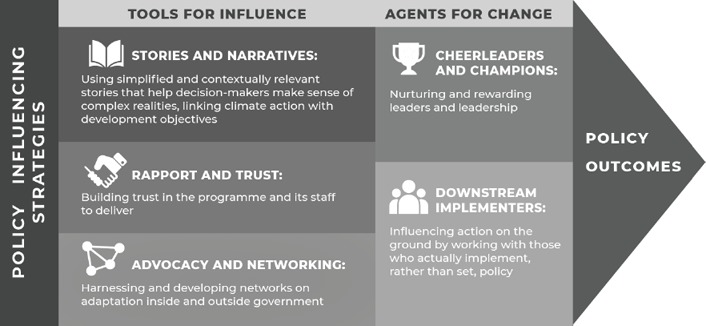
A typology of policy‐influencing strategies **Source**: authors—design by Will Bugler and Anandita Bishnoi (Acclimatise).

This typology, illustrated in Figure 1, illustrates strategies that are generally used in combinations with each other. Points to note are as follows:

- Some of these strategies for influence are more focused on the agents of change, such as the importance of building trust, developing champions or working with those who implement policy down the bureaucratic hierarchy. Others are more linked to the specific tactics used for influence, such as policy narratives, advocacy and networking.
- The different influencing strategies can feed off each other. For example, creating compelling ‘stories and narratives’ becomes a lot easier if you have ‘cheerleaders and champions’ in place who are receptive to and can help shape the narrative.
- There may be trade‐offs to using some of the influencing strategies. For example, reaching out to ‘downstream implementers’ may take a great deal of time and effort, and reduce the chance of nurturing high‐level champions.
- Some policy entrepreneurs are more adept than others at deploying some of these strategies, but teams can look to build capacities that span these techniques.

By examining the use of these strategies in relation to the ACT programme, we can discern some patterns to provide recommendations for the design of technical assistance programmes on adaptation and resilience.

## Policy‐influencing in practice: experience from South Asia

South Asia is no stranger to climate stress and extreme weather events, but climate change is likely to add even greater threats to ecosystems, economic growth, human health, food security, livelihoods and lives (Carabine et al., 2014). Key impacts include flood damage to infrastructure, livelihoods and settlements. Extreme rainfall events, rising sea level and cyclones could combine to cause widespread flood damage, with risks highest in India and Bangladesh (IPCC, 2014). At the same time, an increasing risk of drought may cause water and food shortages. There is also high confidence within climate science of an increased risk of heat‐related mortality, with the region's fast‐growing cities already facing high temperatures. The agriculture sector is particularly sensitive to changes in climate, which will have significant development implications given the high dependence on agriculture for employment in South Asia. Crucially, these risks can all be managed to some extent by additional adaptation actions over and above current levels (IPCC, 2014). Yet adaptation efforts remain constrained by a combination of knowledge, capacity, resources and short‐term political horizons.

This section illustrates policy entrepreneurship and influencing in the context of efforts to mainstream adaptation into institutions and policy processes. It draws on empirical examples from the ACT programme in South Asia, mapping them onto the typology outlined above. It draws particular attention to informal influencing strategies, which are under‐reported in formal ‘projectised’ mechanisms. ACT provides technical and financial support to national governments in Afghanistan, Bangladesh, Nepal, Pakistan and state governments in India in the states of Assam, Bihar, Chhattisgarh, Kerala, Maharashtra and Odisha. It focuses on helping these countries integrate climate change into policies, plans and budgets, as well as strengthening capacity to attract and leverage climate change finance. ACT deploys experts to sit within government units, combined with national and international expertise to provide advice, resources, training and technical knowledge to support systems of planning and delivery.

ACT's theory of change aims to ensure that economic and social systems adapt by integrating climate change into public and private sector policies as well as investments. It does so by shaping and influencing government systems and public policies. In Indian states, the programme was established under a bilateral agreement between the Governments of India and the UK, which has helped the programme establish a working relationship with Indian state governments. In other countries, the programme has relied on strong national and local partner organisations, which in turn have deployed one or more of the five strategies discussed in this article to achieve programme objectives. ACT has a decentralised set‐up whereby a central senior management team guides action and monitors progress but project teams in 12 locations take day‐to‐day strategic and operational decisions.

### Developing adaptation policy narratives

Policy‐makers themselves have suggested that influence requires winning the argument about what the problem is before trying to win the one about what the solution is (Taylor, 2005). Developing and communicating compelling stories or policy narratives is a crucial part of this process. ACT has used an organic process of developing and refining a specific narrative for each location (and/or different parts of the government in each location) on the need for investment in adaptation (see Box 1). This process of ‘domesticating’ climate change issues is contrary to the popular narratives of climate change as an issue dealt with only by scientists or by diplomats at UN inter‐governmental conferences. At the same time, drawing down from the agenda setting of the UN Paris climate change agreement adopted in 2015 can help frame local issues in the context of already‐agreed national priorities.

Early on, ACT carried out internal long‐range planning exercises to identify an initial list of potential actions in each location. These combined the government's political priorities and the ‘entry‐point’ for linking climate change impacts to these actions. This enabled the subsequent technical and capacity support to align with and adjust a wider narrative around development in the location. Thus the narrative was able to build on floods in Assam, water scarcity in Afghanistan and drought in Maharashtra, for example.

The programme regularly monitors prevailing narratives. For example, in Kerala at the start of the programme, climate change was closely identified with the state's long history of environmental and ‘green’ activism, but there was confusion on the distinction between climate change and environmentalism. In Assam, the team compiles a monthly archive from local newspapers that highlights the local impacts of climate change and informs the team's communication with policy‐makers.

A key tactic for building such narratives was to link these risks with areas of government that are best prepared for adaptation action and build out the network and interest from there to reach other areas and actors. ACT has also used a solution‐based narrative to help empower partner governments based on an understanding of what an external support programme could provide. In Maharashtra, ‘climate‐screening’ the government's flagship drought prevention programme provided a positive story about the benefits of incorporating climate change information and adaptation action. This also tried to match the incentive structures in the partner governments, such as by helping leverage additional climate finance to fund priority areas.

**Box 1. Developing adaptation narratives for policy influence in Assam**

In Assam, the absence of policy narratives on climate change acted as both a challenge and an opportunity for ACT's influencing efforts. The initial Assam State Action Plan on Climate Change (ASAPCC) failed to provide a narrative on why it was needed that resonated with the state government. ACT therefore had to convince the government of the purpose and value of the plan, and of action on climate change in general, in terms that aligned with political priorities. In particular, ACT highlighted the link between tackling floods—a recurrent and increasingly severe threat for the state—and the need for action on climate change. This included technical analysis to map vulnerability and the extent of loss and damage in the state. The analysis ACT provided was reinforced by the historically unprecedented monsoon floods of 2017, which had impacts on upwards of 2 million people and resulted in above $400 million in loss and damage for the state. The team leader worked with local journalists to inform their reporting on this and other extreme events to highlight the risks climate change poses of increasing the frequency and severity of flooding.

ACT has maximised policy influence by capitalising on the Chief Minister's own constituency—Majuli Island—being one of the most vulnerable districts to climate change and is of significant cultural and religious importance to the state. First, the programme carried out an assessment of community‐level risks in the district, which was released by the district administration. This helped highlight the human dimension of climate change and also attract media and political attention.

Another key strategy for ACT in Assam has been to work with sectoral departments on climate change‐related targets in the state government vision document for achieving the Sustainable Development Goals (SDGs), developing a digital interface that enables simultaneous tracking and reporting for both SDG and ASAPCC targets. This links climate change as part of the development progress narrative, helping the government mainstream expenditure for action on climate change.

**Source**: authors.

In Pakistan and across a number of states in India, value chain analysis uses a positive story of delivering livelihood benefits by identifying crops that are climate‐resilient, environmentally friendly and suited to local production and market systems. For the state of Punjab in Pakistan, such analysis on canola, mung beans and chickpeas has revealed bottlenecks and generated recommendations to government, such as on crop‐specific sowing and threshing machines, that will maximise productivity and income from these more climate‐resilient crops.

One challenge of relying on stories and narratives to influence policy change on climate change lies in ensuring ‘unpopular’ issues do not get ignored. For example, across all locations, government interest in accessing additional sources of climate finance has been a useful ‘positive’ entry‐point, but moving the discussion on to how governments can maximise the adaptation benefits from their domestic budget has been a slow and difficult process. In addition, focusing only on issues where there are positive solutions can mean that issues for which the solution is not easy to implement, or not politically viable, do not get the attention they may deserve (such as soil degradation, water pricing).

### Building rapport and trust

Building trust with government officers and politicians is fundamental to an externally supported programme's ability to influence policy, but it can be both difficult and‐time consuming (Brouwer and Biermann, 2011). The ACT programme co‐located team leaders and technical experts within government units with a formal mandate for climate change coordination, or otherwise located them nearby, visiting the government office regularly (often daily). This contrasts with other models of technical assistance that either base the technical team centrally in the region or send in international advisors for short‐term assignments. Much of the reported influence comes from rapport and trust built up through the day‐to‐day contact with government officials, which also helped to give the programme wider credibility. Informally, ACT staff have provided advice and policy inputs on topics beyond the formal purview of the programme strategy. As a result, when a window of opportunity for developing adaptation policy has opened, ACT has been the visible and trusted first port of call for assistance.

For example, in Chhattisgarh, India, ACT has supported the creation of the State Centre for Climate Change as part of the government structure. Upon the Centre's inception in 2016, ACT operations moved inside it, strengthening everyday interactions with government and its decision‐making structures on climate change. The permanence also helped give ACT programme staff the time and opportunity to push policy documents over the necessary procedural hurdles to achieve sign‐off. In Maharashtra, having a technical expert located full time within the government secretariat proved crucial for the long process of getting a Climate Change Policy drafted, approved and adopted by constantly raising the issue with relevant officials and managing the internal bureaucracy.

Building an informal rapport with senior bureaucrats is crucial to ACT's successful policy influence. The personalities and experience of the local ACT teams have determined their level of success in having strong working relationships with their government partner. They often have prior experience with the relevant government departments and their key actors, and know the local policy context and its dynamics. As policy entrepreneurs, the ACT staff often looked initially for common connections between the team and officials—for example whether they had had similar academic training. For example, personal trust improved in one case when it came to light that the team leader's sibling had trained at university with the climate change nodal officer.

Another key tactic that ACT uses to build trust and rapport is to organise events for government counterparts without putting ACT in the forefront. For example, a high‐profile Climate Change Conclave in Chhattisgarh in 2017 raised the profile of the government with the invited experts from across the country. As a result, the government then trusted that the local team would try and make them look good. Similarly, most technical outputs from the programme are branded as the government's, with limited (if any) recognition of the contribution of the programme and the donor.

Nevertheless, embedding within the government machinery poses some risks, as embedded technical advisors could be co‐opted into other work of the department rather than focusing on the agenda of the programme. There is also an unfortunate reality that certain politicians and bureaucrats may accept or ‘trust’ some types of people more than others—depending on gender, age, religion, where they are from or their disciplinary background, among other things. For example, while embedding helped build trust in some cases, in others the programme was able to influence more effectively by drafting in nationally or international recognised expertise at strategic points.

Investing time and resources in trust‐building can also be a risk, as relations that have taken time to build can be nullified by periodic change in officials or in the government ruling party, as has occurred in Indian states of Kerala and Assam during the ACT programme lifetime. In part to mediate this risk of bureaucratic ‘churn’ and political change, a policy‐influencing tactic common to many of the ACT locations has been the engagement of researchers and organisations that already have links with and are trusted by political and bureaucratic decision‐makers. In Odisha, the programme worked with a local organisation (CTRAN), which had a track record on adaptation in the state and understood the policy space, having provided support since the earlier inception of the SAPCC. Similar tactics were employed in Bihar (see Box 2). Again, however, the tactic of engaging established consultants carries a risk of implicitly restricting newer, more competent or more innovative consultants and researchers who might bring new perspectives, ways of working or efficiencies.

**Box 2. Trust and policy influence in Bihar, India**

In Bihar, efforts by ACT to strengthen the state government's capacity to manage and finance action on climate change initially drew on an expert consultant from outside the region, but the issue did not resonate strongly with the government's existing interests and priorities. The programme team thus decided to engage the Bihar‐based Asian Development Research Institute (ADRI), which had credibility with the Government of Bihar on social, political and economic issues but no significant prior experience of working on climate change. This credibility had been cemented through strong professional relationships between ADRI's senior leadership and the state bureaucracy and politicians as well as through the vast amount of support that ADRI was providing to the Government of Bihar on designing, delivering and monitoring development initiatives. The ACT local team has supported ADRI in technical analysis, reports and conferences on climate change issues in the state and to build its internal capabilities. Establishing ADRI as a long‐term state knowledge partner on climate change under the Government of India's Knowledge Management Mission on Climate Change will help sustain the technical support and influence of ACT.

**Source**: authors.

### Advocacy and networking to mainstream adaptation

ACT has helped develop networks external to government that can generate analysis, share knowledge and provide pressure and legitimacy for adaptation policies and actions. In Afghanistan, ACT helped the Climate Action Network South Asia (CANSA) provide climate change campaign training to a group of Afghan civil society organisations (CSOs) working on environmental causes. This group was subsequently recognised as Afghanistan's first National Steering Committee of CANSA, giving them a voice and platform. This helps to ensure these CSOs can work as an informal pressure group to enable continued government action on adaptation even after ACT funding ends.

ACT has also engaged the media to enhance influence, organising high‐profile events to launch government policy documents. For example, in Bihar, the Deputy Chief Minister launched a technical report on financing of adaptation at a high‐profile event that received significant media coverage. By linking to wider news stories and events it is easier to generate media interest. For example, in Pakistan, ACT provided technical support to integrate climate change into the first‐ever Punjab Women Development Policy, which was launched on International Women's Day and received considerable media coverage. In Afghanistan, ACT has facilitated training to journalists on climate change reporting and built a network of interested reporters to help with this influencing strategy. In Bangladesh, this influence extends to accountability, with ACT planning to train a group of non‐governmental organisations, think tanks and journalists in climate budgeting to support an advocacy network that can scrutinise government spending on adaptation.

A different tactic has been to facilitate climate change communities of practice within government. The nominated focal point for climate change commonly lies within the environment and/or forestry department, which are often weaker in their ability to influence adaptation policy in other important sectors such as water or agriculture (Klein et al., 2007; Rai and Fisher, 2016). ACT has built opportunities for networking and for champions within government to ‘advocate’ internally—for example establishing a series of thematic working groups involving government and non‐government actors to develop Nepal's National Adaptation Plan (NAP); supporting the Climate Change Cell in Chhattisgarh to act as a knowledge centre and organising seminars, workshops and trainings for different departments; and facilitating cross‐sectoral climate change committees across a range of locations. The engagement of networks of citizens and communities can also help support policy‐influencing objectives. ACT has supported a number of stakeholder engagement processes to inform decision‐making of adaptation policy—for example as part of the Nepal NAP process, although it has proven difficult to get sufficient representation and inputs from the most vulnerable and excluded. In Maharashtra, ACT is supporting the state government's efforts to build a network of 4,000 ‘water champions’ as part of its Water Literacy Campaign. As with journalists and research networks, engaging citizens can also put pressure on government in relation to adaptation policy and activities, especially by promoting accountability, including for the spending of climate finance (Schalatek, 2012).

**Box 3. Developing a cross‐government climate network in Kerala**

Networks within government can be important means of enhancing the influence of the agency responsible for managing climate change—extending its reach across sectors and also opening up channels for informal influence. In Kerala, climate change was not a high policy priority for the government when ACT began. A (central government‐mandated) SAPCC had been drafted but funds were not allocated for its implementation, and the climate change lead department did not hold significant power across government.

One of many strategies used by the local ACT team to increase political commitment on climate change has been to strengthen the influence of the Climate Change Cell within the Environment Department over the line departments. ACT worked with the Principal Secretary of the Department to ask other secretaries to nominate departmental nodal officers for climate change. Many now have a three‐member focal team, helping mediate the risk of staff churn and institutionalising climate change within the state's planning and organisation processes. This provides the Climate Change Cell with a direct avenue to communicate with the line department, to understand and inform its policy processes. All letters to the respective department on climate change are sent by/from these officers, and they represent their department at meetings on climate change.

These individuals have established a network and more informal community of practice, sitting across government departments, universities, institutions and other government agencies. An email and WhatsApp group has provided an informal platform for discussing new ideas, sharing best practices and building relationships across the group. This has helped overcome the culture of hierarchy, which often prevents free and open communication between and within different departments.

**Source**: authors.

Working through networks of other partners and individuals does affect the ability to control the policy‐influencing process directly. While ACT has been able to build these networks’ level of understanding and capacity, there has been no certainty on what they will eventually advocate for. It is also a more indirect, and less immediate, process of influencing, and the contribution of the programme to achieving the end result can be difficult to measure. For example, the highly participatory process used to prepare Nepal's NAP, involving all climate‐relevant ministries and agencies, meant the process took a lot longer than it would have if only a single department, or a consultant, had drafted the document.

### Supporting cheer‐leaders and champions

Nurturing and supporting people who can champion adaptation and mainstreaming is crucial to securing lasting institutional and policy change (Meijerink and Stiller, 2013). In many locations for ACT, opening the door to policy influence has hinged on securing the interest of a high‐profile champion. In some cases, this has been a senior politician. For example, the Deputy Chief Minister in the Indian state of Bihar became a key ally of the programme, while in Pakistan an advisor to the programme is located within the Prime Minister's Office as a focal person for climate change. In others, it has been a senior official. For example, the Secretary of the Water Resources Department in Odisha, who had a background in disaster management and recognised the need to address flooding issues, has championed important work with the programme. In Assam, prior working relationships of the team leader helped the programme to engage the Chief Secretary to the Department of Environment and Forests, where he was able to make the case for Assam showing leadership on adaptation. This buy‐in helped the programme move forward far more efficiently, with the Chief Secretary dealing with the bureaucratic hurdles from inside the system.

In Afghanistan, the main entry‐point has been through mainstreaming climate change into the Natural Resources Management (NRM) Strategy. ACT identified and supported the Director General of NRM within the Ministry of Agriculture, Irrigation and Livestock as a champion of integrating adaptation. A policy window was opened for integrating adaptation into a draft version of the strategy when ACT was invited onto the technical working group and, along with other partners, such as UN agencies and the World Bank, worked to support government officers to develop mainstreaming actions within this strategy. In Nepal, in developing the workstream on adaptation and tourism, the focal point and the nodal officer had both previously been posted in the Ministry of Environment, where they had had substantial exposure to the issue of climate change adaptation. As a consequence, they could relate the problem of climate change to tourism and influence take‐up of the issue in the Department of Tourism.

In some cases, fostering these champions has meant being highly responsive to the demands and interests of senior figures, orienting the programme's activities to those areas of adaptation where there would be sufficient leadership to push policy change and implementation. In Bihar, the programme took up the topic of siltation because of the strong demand and interest from the Principal Secretary, despite the link to climate change not being straightforward. The programme has been able to quickly move forward the government's thinking on siltation, including through a Sediment Management Framework, but the goodwill generated also helped progress other areas of work.

There are, however, more critical perspectives on working with champions. Relying on individual champions within government may privilege the interests of senior politicians and bureaucrats over those that might be generated through more participatory consultations or bottom‐up assessments. In addition, the programme has to manage expectations and requests for personal interests and incentives that may be beyond the programme's remit. In general, working only with influential actors risks not being able to challenge the *status quo*. These individuals and actors, who already have ‘power’ within the current system, are unlikely to champion issues that could challenge the systems and structures within which they operate. For example, in some locations, it was difficult to get interest from ‘champions’ to look explicitly at water‐intensive or unsustainable crops such as sugarcane, given that these farmers are important political lobbies.

For many locations in the ACT programme, influence has also been sought indirectly by identifying and working with other actors who have the most influence on policy‐ and decision‐maker networks. In Kerala and other locations, ACT hired as an advisor a former bureaucrat with strong networks in the government system, to help push files through and gain access at the highest levels. In all locations, ACT tries to use local experts, researchers and organisations to deliver the technical workstreams, who themselves have networks and relationships within government that the programme can utilise. In Nepal, working with a local partner, Practical Action, meant ACT could build on its pre‐existing networks, which in turn helped position it in what is a busy field of national and international organisations and programmes. In Maharashtra, previous work contacts helped to persuade the British High Commissioner to raise the urgency of signing off the SAPCC when meeting the state's Chief Minister.

### Downstream implementers

Many governments already had some form of climate change policy framework in place when ACT support started but recognised that there was a significant lack of understanding and action on implementation. In addition to working with and building high‐level ‘champions’ within the government, ACT has therefore also worked with the ‘implementers’ within the bureaucracy. In some cases, ACT did not immediately have an entry‐point at the senior official or political level. For example, in Chhattisgarh, ACT started working with medium‐level officials to build the programme's credibility to then allow it to engage at a higher level. In all locations, ACT has made a targeted effort to quickly move its engagement from the responsible agency for climate change (such as environment departments or ministries of climate change) to the sectoral agencies that can inform adaptation on the ground.

In Afghanistan, mid‐level bureaucrats were the main point of influence, precisely to take the messages both up and down the hierarchy. In Odisha, while the senior bureaucrats in the Environment Department were consulted and were the main point of coordination for the programme, ACT worked primarily with technical staff in the Water Resource and Agriculture Departments on flood management and mainstreaming adaptation into the government's water resource and agriculture planning systems. In Nepal, during the recent transition to federalism and related government upheaval and uncertainty, ACT focused its engagement on officials and actors who were not directly affected, such as the Central Bureau of Statistics. In all locations, offering targeted training, particularly in connection to developing climate finance proposals, has proven an effective way to engage and influence officials lower down the hierarchy. This includes relatively technical officers, who often do not get the opportunity to participate in climate change‐focused events and workshops, or many opportunities to build their own profile within their departments.

The focus on working with implementers is also supported by the development of pilot activities. Frequent calls from government for pilot activities reflect their desire to see more implementation, helping demonstrate how policy change works on the ground and acting as a valuable influencing strategy. While ACT cannot support all activities requested, given its limited budget and mandate to provide technical assistance, some activities, such as support to pilot an integrated irrigation and agriculture planning framework in Angul district in Odisha, have helped to secure wider policy influence.

One challenge of working directly with ‘implementers’ within the bureaucracy is that they usually lack the authority to take decisions and make change happen quickly. For example, in India, the programme has found that bureaucrats from sector‐focused cadres can sometimes find it difficult to influence bureaucrats from the central administrative cadre, even if they are at the same level of seniority. In most locations, working with an ‘implementer’ within the bureaucracy who has the visible backing and support of a senior officer has been the ideal situation. In addition, lower‐level officials are often easier to access and engage but are usually more risk‐averse. They tend to be hesitant to take actions beyond the scope of their responsibilities and job description and prefer to follow clear guidance and instruction from above.

## Conclusions: implications for adaptation and technical assistance programmes

Despite the considerable critiques and challenges of resilience approaches, operational approaches to building resilience have grown and are championed by major donors hoping to reduce humanitarian spending and protect development gains (Weichselgartner and Kelman, 2015; Tanner et al., 2017). The Paris climate change agreement of 2015 has revitalised a growing global effort to adapt development policies and plans to a changing climate. Creating and implementing NAPs will require a significant upscaling of efforts to mainstream adaptation concerns into development, with many countries requesting technical support from the international community. However, many of the transferable lessons to date on how best to support mainstreaming processes have been in the form of technical approaches such as risk assessments and toolkits. In contrast, this article has provided an empirically informed review of some of the more tacit and informal approaches used to influence adaptation policy, particularly highlighting the role of individual policy entrepreneurs in these processes.

In doing so, this article provides some lessons for emerging critical thinking in climate adaptation and resilience‐building. The research suggests that adaptation mainstreaming needs reframing away from a series of technical challenges and towards an understanding that the construction and negotiation of climate change leads to different distributional implications for impacts and adaptation. It asks whether the models of governance and theories of change assumed by a predominantly technically led framing of adaptation mainstreaming are appropriate. Given the concentration of political and bureaucratic power and informality of many policy processes found in many administrations, it questions whether the dominant technical models that underpin externally supported mainstreaming efforts are reflected by, or even compatible with, the realities of governance in many places. Drawing on the examples presented here and foregrounding the importance of informal and tacit strategies for policy influence can therefore help others designing and providing technical assistance to support national and sub‐national governments to mainstream adaptation into their policies. These programmes can factor in design elements in order to maximise this potential, while also balancing and developing the skill sets of staff to cover the different types of policy influencing tactics presented here.

First, many of the strategies outlined in this article require a thorough understanding of the changing policy context, especially locally. One way to promote this understanding is to engage programme staff who have prior experience with the relevant local bureaucracies and adaptation issues. However, programmes can also undertake regular analysis of the changing political economy influencing adaptation mainstreaming, including assessments of both proximate and more distant policy drivers. Bringing staff and partners into the process of collating these analyses promotes buy‐in to enable politically astute programming and better tracking of the intervention's contribution to change processes.

Second, a more decentralised approach to programming helps to ensure that approaches to policy‐influencing are locally appropriate and support local capacities. Decentralisation can also help to empower communities and local governments by increasing local autonomy, create mechanisms for cross‐sector and cross‐scale information‐sharing among decision‐makers and improve accountability of local decision‐makers (Agarwal et al., 2012). For a technical assistance programme, this is more likely to be effective where team leaders in different locations have greater ability to define programme priorities and methods rather than acting only as implementers of a centrally determined strategy and approach. Political economy assessments can help inform this process, including by gauging the pre‐existing level of engagement on climate change issues (Cooke, 2018).

Third, policy‐influencing strategies may also benefit from the iteration and flexibility of an adaptive management approach to programming. Adaptive management is an intentional approach to making decisions and adjustments in response to new information and changes in context (USAID, 2018). Crucially, this is not about changing goals during implementation, but about changing the path being used to achieve the goals in response to changes in the policy and programming context. Setting up a programme to regularly revisit activities and outputs will enable policy entrepreneurs to learn as they go and to take advantage of unexpected opportunities for policy‐influencing as they arise. Experience from adaptive management of social‐ecological systems demonstrates the challenge of operating when outcomes are unclear (Armitage et al., 2015). However, the real challenge is to establish programmes that are ‘committed to experimentation and “learning by doing” from the start, as distinct from building in flexibility to respond to changes in circumstances during implementation’ (Wild et al., 2017, p. 23).

Adaptive management approaches may also widen the opportunities for more politically informed approaches, acknowledging that the drivers of change are often rapidly changing themselves (see Box 4). As such, more in‐depth ‘development entrepreneurship’ methods have been proposed that explicitly and iteratively integrate politics into project and programme implementation to find technically sound and politically possible reforms (Faustino and Booth, 2014; Cooke, 2018).

Fourth, the design and monitoring and reporting processes of technical assistance programmes need to recognise the large amount of ‘off‐terms of reference’ work that accompanies informal influencing strategies. Explicitly writing staff time and resources into programme plans for influencing can acknowledge the scale of efforts required by policy entrepreneurs to influence more effectively. One way to facilitate such processes is for design and reporting formats to emphasise outputs and outcomes rather than activities. At the same time, care is needed so that monitoring captures learning on successful and unsuccessful strategies for policy‐influencing (Dinshaw et al., 2014).

**Box 4. Employing an adaptive approach to programming in Nepal**

The ACT programme in Nepal started with an initial set of activities identified through a long‐range planning exercise. Many of those activities had to be dropped or abandoned halfway through owing to changing context and government priorities. ACT's support to the NAP formulation process similarly had to be reoriented when the government received a large ‘readiness’ grant from the Green Climate Fund for the same purpose. Furthermore, Nepal has seen a significant political upheaval during the lifetime of the programme, including changes in government, adoption of a new constitution, wide‐ranging decentralisation of power to newly‐formed provincial and local authorities and phased elections for local, provincial and national government. In addition, the country has a very crowded climate policy sphere where identifying innovative solutions is a daunting task in itself.

The programme has hence had to take an adaptive approach to be able to adjust the work and achieve the intended results. The programme team had to be constantly entrepreneurial and innovative to identify new entry‐points for influence. ACT focused on sectors and issues that required climate mainstreaming but were less affected by the contextual challenges. Using existing relationships, the programme reached out to ministries such as the Ministry of Culture, Tourism and Civil Aviation and the Ministry of Urban Development and presented a contextualised narrative on the need for mainstreaming climate change into these sectors. The programme also engaged with the private sector working on tourism to mainstream climate change adaptation within their investments.

Deploying this adaptive approach in Nepal was possible in large part because of the flexible delivery model and monitoring and reporting framework inherent in the programme.

**Source**: authors.

The design of programmes can make explicit space for private, informal interactions between the team and local stakeholders that can be fundamental to building trust. This may entail budgeting time explicitly within workplans to be used flexibly and adaptively as required, including for building relationships with key individuals and organisations within and outside government. Such programming can also create space for understanding and promoting informal and shadow networks to develop social relationships. Acknowledging the importance of these networks may also require rethinking the personal skills and working routines that are incentivised within organisations (Pelling et al., 2008; Armijos et al., 2017). As such, institutionalising mainstreaming requires directing attention and resources specifically to the skills and strategies of the government and other local champions to influence policy without the benefit of external technical assistance.

Finally, policy entrepreneurship can be fostered by setting up programmes to be able to respond to unexpected opportunities and policy ‘windows’ that open rapidly but often close just as quickly, thereby requiring rapid action (Birkmann et al., 2010). Programmes therefore need to be proactive in supporting future potential work areas that are ready for these windows to open. This can be promoted by creating specific funds within programmes that are available for such opportunities. Even when these are in the wider remit of climate change action rather than strictly in adaptation, the ability to rapidly and flexibly mobilise resources can be crucial to building trust in the programme and its staff.

## Acknowledgements

The authors would like to thank the entire ACT programme team for providing the experience and learning to inform this paper, in particular Nirmala Sanu, Soumik Biswas, Pankaj Kumar, Rishu Garg, Naman Gupta, Arif Pervaiz, Azim Doosti, Nirmala Sanu and Vidya Sounderajan for sharing their experiences and insights. The research benefited from early conversations with Simon Maxwell. Special thanks go to Emily Wilkinson (ODI), Cristina Rumbaitis del Rio (ACT) and two anonymous reviewers for peer review comments; thanks to Uma Pal for editorial support.

The opinions expressed in this article are those of the authors and do not necessarily represent the views of the UK Department for International Development, which funds the ACT programme.
